# Evaluation of Korean methane emission sources with satellite retrievals by spatial correlation analysis

**DOI:** 10.1007/s10661-024-12449-w

**Published:** 2024-02-22

**Authors:** JunGi Moon, Changsub Shim, Jeongbyn Seo, Jihyun Han

**Affiliations:** 1 Korea Environment Institute, Sejong, South Korea; 2 Pusan National University, Busan, South Korea

**Keywords:** Methane, South Korea, TROPOMI, Emissions, Spatial correlation

## Abstract

Methane is a significant greenhouse gas (GHG), and it is imperative to understand its spatiotemporal distribution and primary sources in areas with higher methane concentrations, as such insights are essential for informing effective mitigation policies. In this study, we employed TROPOMI satellite retrievals to analyze the spatiotemporal patterns of methane distributions and identify major emission sources in South Korea over the period from August 2018 to July 2019. Additionally, we examined the spatial correlations between satellite methane retrievals and emission sources to characterize regions with higher methane levels on an annual basis.

Concerning spatial distributions, concentrations exceeding 1870 ppb were predominantly observed in western non-mountainous regions, particularly in rice paddy areas. Moreover, sporadic concentrations exceeding 1880 ppb were detected in large ports and industrial zones, primarily located in coastal regions of South Korea.

Our spatial correlation analysis, conducted using the SDMSelect method, identified specific emissions contributing to regions with higher methane concentrations. There were some areas with relatively strong correlations between high XCH_4_ and emissions from the domestic livestock industry, fossil fuel utilization (specifically, the oil and gas sector), landfills, and rice paddies. This analysis, incorporating domestic emission inventories and satellite data, provides valuable insights into the characteristics of regional methane concentrations. In addition, this analysis can assess national methane emissions inventories, where there is limited information on the spatial distributions, offering critical information for the prioritization of domestic regional policies aimed at reducing greenhouse gas emissions.

**Supplementary Information:**

The online version contains supplementary material available at 10.1007/s10661-024-12449-w.

## Introduction

Methane (CH_4_) is one of the six greenhouse gases (CO_2_, CH_4_, N_2_O, SF_6_, HFCs, and PFCs) identified in the Kyoto Protocol, and is known to be responsible for about 30% of global warming (IPCC, 2021). The global warming potential (GWP) of CH_4_ is 84 times higher than CO_2_, and it has a short residence time (about 10 years), meaning that reducing methane emissions can have a relatively instant impact on global change (Bousquet et al., 2018; Prather et al., 2012; Zhao et al., 2019). Therefore, the importance of accurate monitoring and reduction efforts is increasing (IPCC, 2014; Skytt et al., 2020; IPCC, 2021).

Since the late twentieth century, atmospheric methane concentrations have steadily increased. After a stagnant period between 2000 and 2006, methane has increased again and has rapidly increased since 2014 with the largest increase (19.94 ppb/year) observed in 2021 (IEA, 2021; Yin et al., 2021; Butler and Montzka, 2016; Peng et al., 2022).

Methane is mainly emitted from natural sources (wetlands, inland water, termites, etc.) and anthropogenic sources (fossil fuel uses, agriculture, waste, etc.) (IPCC, 2021). Atmospheric methane is mainly removed through chemical reactions with OH radicals in the atmosphere, but recent imbalances between emissions and removals may have caused an increase in methane concentrations (Peng et al., 2022).

The imbalance in the methane budget can result from a combination of three factors: anthropogenic and natural sources and OH radical decrease in the atmosphere, but the exact amounts and the causes have not yet been identified (Rigby et al., 2017; Nisbet et al., 2019; Turner et al., 2019; Rosentreter et al., 2021).

For example, in the bottom-up approaches, the methane emission between 2008 and 2017 was about 737 Tg/year, while the averages of top-down approaches calculated it to be 576 Tg/year, with a discrepancy of 28% (160 Tg/year) compared to the top-down method (Saunois et al., 2020). The differences are mainly attributed to the high uncertainty of the bottom-up emission inventories (Cheewaphongphan et al., 2019), and researches suggested that the top-down methane emission inventory in East Asia may have been overestimated (Bergamaschi et al., 2013; Thompson et al., 2015; Patra et al., 2016).

Methane emissions are largely generated from natural sources such as wetlands, inland waters, geological sources, termites, and oceans. Those make estimating emissions more difficult, contributing to the uncertainty of the bottom-up methane emission inventory (Saunois et al., 2020).

To efficiently reduce regional methane emissions, it is necessary to identify high-concentration emission areas and prioritize reduction efforts based on reliable emission inventories. However, methane is primarily emitted in a diverse manner, making it challenging to accurately monitor and quantify emissions. Therefore, it is necessary to verify existing methane emission inventories and to identify the sources of high-concentration areas.

This study aims to verify methane emission inventories in South Korea and identify the main sources of high-concentration areas. Using TROPOMI XCH_4_ satellite data from August 2018 to July 2019, the spatial distribution of methane concentrations was analyzed and high-concentration areas were identified, which can provide information for domestic greenhouse gas reduction policies.

## Data and method

### TROPOMI data

The TROPOsphere Monitoring Instrument (TROPOMI) sensor, mounted on the Sentinel-5P satellite launched by the European Space Agency (ESA), has been producing and providing daily observation data on atmospheric pollutants such as nitrogen, sulfur dioxide, and ozone since its launch in October 2017. TROPOMI has a wide observation coverage of 2600 km, allowing for global daily observations, and its high spatial resolution of 7 × 7 km^2^ enables not only global but also regional scale air quality monitoring. In the case of methane, data with a spatial resolution of 5.5 × 7 km^2^ has been provided since August 6, 2019, with a finer spatial resolution (Lorente et al., 2021; Veefkind et al., 2012).

In this study, we used TROPOMI XCH_4_ daily data from August 2018 to July 2019 provided by GES DISC (https://tropomi.gesdisc.eosdis.nasa.gov/data/S5P_TROPOMI_Level2/S5P_L2__CH4___.1/) to analyze the distribution of methane concentrations in South Korea and we sampled those retrievals to a 0.1° × 0.1° spatial resolution to make spatial correlation analysis with other emissions data.

The TROPOMI XCH_4_ data have been validated with global Total Carbon Column Observing Network (TCCON) observational data, exhibiting an average bias of approximately 0.68% (Sha et al., 2021). Although there has been no specific validation conducted in South Korea, reference can be made to recent validation results in the nearby region (Saga, Japan), which showed a bias of − 0.9% (− 17.6 ppb, Lorente et al., 2021).

### National emissions data

According to the “National Greenhouse Gas Inventory Report” published by the Greenhouse Gas Inventory and Research Center (GIR) in Korea, there are four major sectors of methane emissions: fossil fuel use (8.7% of the total emissions), livestock farming (21.3%), rice paddy (22.7%), and waste landfill (28.2%). Emissions from fugitive processes were excluded due to the lack of direct measurements and spatiotemporal information. Excluding fugitive processes, 94% of all current methane emissions in South Korea belong to these four sectors (GIR, 2020).

In this study, we used indicator data that can represent each emission of the four fields to analyze their correlation. The indicator data were not intended to accurately measure the exact amount of methane emissions but rather represent the variability of activity levels according to their spatial variations. Correlation analysis mainly represents relative changes in values, so indicator data were instead of absolute values of methane emissions. There are four indicator data selected: gas and petroleum consumption for the energy sector (hereafter “fossil fuels,” ammonia emissions for the livestock sector (hereafter “livestock”), rice paddy acreage for the agricultural sector (hereafter “rice paddy”), and landfill waste for the waste sector (hereafter “landfill”).

In the case of fossil fuel use, only the usage of petroleum and gas, excluding coal usage and associated emissions, for each region was used to estimate methane emissions from the fossil fuel sector presented in the “Regional Energy Statistical Yearbook” by the Korea Energy Economics Institute (KEEI, 2020). The methane emissions in the fossil fuel sector were estimated using the methane generation calculated based on the emission factors (0.15 kg CH4/TJ (petroleum), 0.41 kg CH_4_/TJ (gas)) and unit conversion factors (0.0419 TJ/Toe) presented in the “National Greenhouse Gas Inventory report of Korea 2020” by the Greenhouse Gas Inventory and Research Center in Korea (GIR, 2020).

For estimations from the livestock sector, ammonia emissions by county were obtained from the CAPSS (Clean Air Policy Support System), provided by the NAIR (Korean National Air Emission Inventory and Research Center). The CAPSS ammonia emissions for livestock were calculated by applying the head of livestock (cattle, pigs, chickens, etc.) and emission factors, and were assumed to be representative values for methane emissions in the livestock sector (Lee et al., 2011).

Methane emissions in the agricultural sector primarily occur during rice cultivation. Thus, rice paddy acreage was used as an indicator to estimate methane emissions in the agricultural sector. The rice paddy acreage data was obtained from the annual crop production survey results provided by Statistics Korea, and the amount of methane emissions was estimated using the production amount of paddy rice (92.9% of the total) by county (KOSIS, 2023).

In the waste landfill sector, it is known that methane is generated as a result of the anaerobic decomposition of landfilled waste. Therefore, the amount of waste landfill per facility (ton/year) by region, which is provided in the nationwide waste generation and treatment status by the Ministry of Environment (MOE), was used as data to estimate methane emissions in the waste landfill sector (MOE, 2019).

**Fig. 1:**
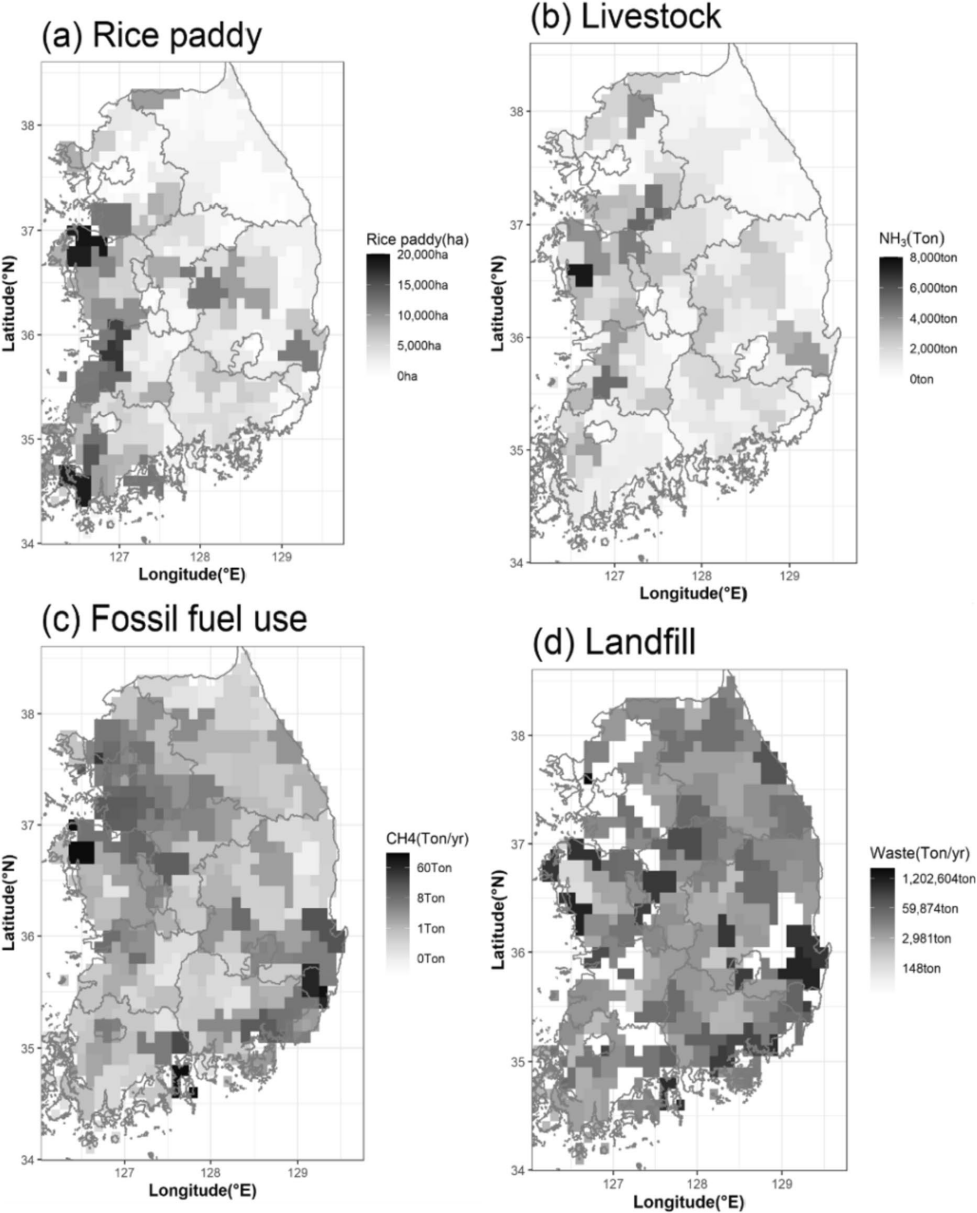
The spatial distributions of (**a**) rice paddy, (**b**) livestock, (**c**) fossil fuel use, and (**d**) landfill over South Korea were described in the “National emissions data” section

The purpose of utilizing these national statistics is to ensure the accuracy of the spatial correlations with the satellite data by incorporating official county-level information into the indicators. We focused primarily on the spatial characteristics of the methane concentrations driven by the predominant sources. The spatial distributions of the four emissions analyzed for this study are shown in Fig. 1.

Currently, those emission indicators do not have gridded information, providing annual emissions for each county level. We transformed the county-level data into geo-gridded data with the same resolution (0.1° × 0.1°) as the TROPOMI data and used it to analyze the characteristics of areas with higher methane concentrations.

The names, administrative district code (denoted by sig_CD), and maps of the counties used in the analysis process are attached in the supplementary (Figure S-1).

### Method

While the XCH_4_ can be influenced by variables such as atmospheric transport and chemical reactions, the analysis of spatial correlations was conducted on the assumption that the primary emission sources predominantly influence the annual methane concentration distributions.

In this study, the spatial correlation of two raster data (the TROPOMI satellite data and 4 emissions datasets) was evaluated through correlation analysis between groups of pixels (area) and their variation, which is distinguished with the analysis based on a single pixel.

To calculate the spatial correlation, species distribution modelling (SDM) in SDMSelect, R (v.3.5.1) was used (R Core Team, 2018; Rochette, 2017). SDM is a widely used technique in spatial analysis, particularly in ecological and spatial conservation fields (Araújo and Peterson, 2012; Naimi and Araújo, 2016; Rochette, 2017).

**Fig. 2:**
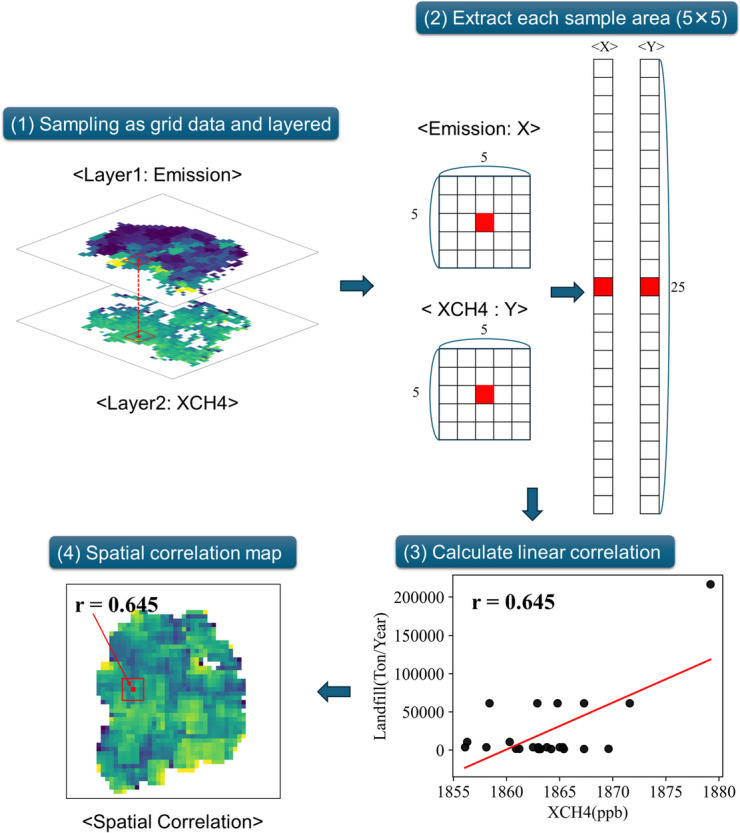
The conceptual schemes of processing the species distribution modeling (SDM)

Here are descriptions of the SDM process applied (Fig. 2). First, the two datasets (XCH_4_ and emissions) were prepared with the same array format. To convert all emissions data to 0.1° × 0.1° gridded data, the geogrid data were extracted and overlapped with a feature class at the level of cities and districts. Using the Conversion Tool in ArcMap (10.1), the overlapped feature classes and emission sources by city and district were matched and converted to a raster format for the grid. The conversion includes the process of spatial representation, value assignment, and interpolation and the tool ensures accuracy (https://pro.arcgis.com/en/pro-app/latest/tool-reference/conversion/feature-to-raster.htm). Only the grids where both TROPOMI data and emission source data are available were prepared and used for the analysis, excluding the grids with high uncertainties in the island and coastal areas.

Second, we extracted the focal area (sampled area). The structure of the focal area was set to 5 × 5 grids (25 grids) through repeated analysis (Fig. 2). Third, we calculated spatial autocorrelation between two datasets in the same location and the focal area in the overlapped two data and repeated this process to cross-validate the entire grids. The spatial autocorrelation is applied with the assumption that the values of variables sampled at nearby locations are dependent on each other (Elith & Leathwick, 2009). Lastly, the correlation coefficients between the satellite data and emission dataset were visually represented for the grid located at the center of the focal area to create a spatial correlation map. Since all the emission indicators are annual data, the spatial correlation analysis was conducted on an annual basis.

## Results

### Spatial distributions of XCH_4_ and the characteristics of higher methane concentrations

**Fig. 3:**
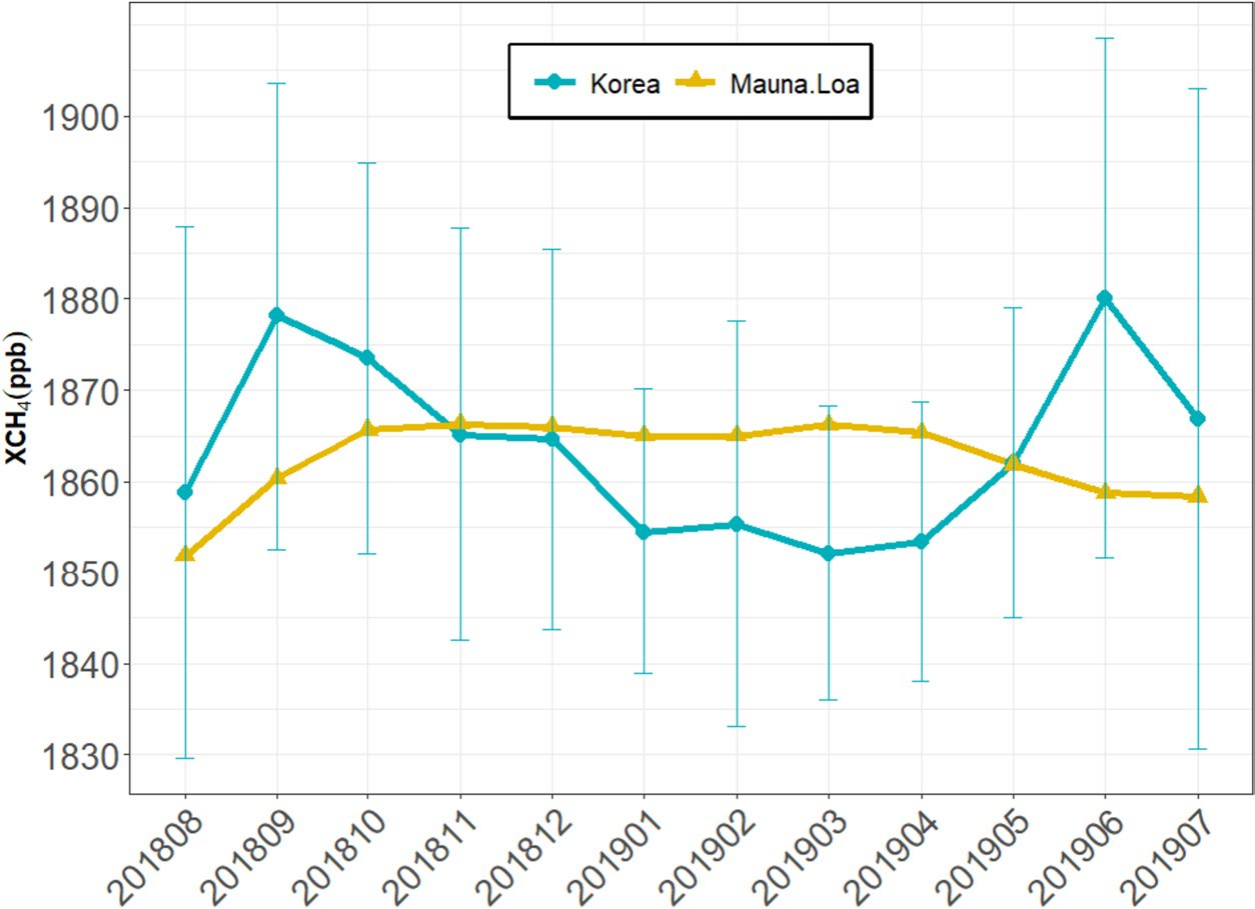
The monthly mean variabilities and standard deviation of XCH_4_ over South Korea (blue line) and Mauna Loa in Hawaii (global northern hemispheric background (yellow line)

Using TROPOMI data, monthly methane concentrations (XCH_4_) in Korea were sampled from August 2018 to July 2019. Figure 3 shows the monthly variations of XCH_4_ in South Korea, indicating a decreasing trend from October 2018 to March 2019, followed by an increasing trend from April onwards (Fig. 3). Unlike the TROPOMI of Mauna Loa, which often represents the northern hemispheric background, Korea had higher methane concentrations in the summer than in the winter (Fig. 3). This can be explained by higher methane concentrations from rice paddies and wetlands in the Asian region during the summer (Hayashida et al., 2013; Ito et al., 2019).

During the study period, the average XCH_4_ in South Korea was approximately 1858 ppb, which was similar to that of Mauna Loa Observatory (1863 ppb) during the same period. The annual maximum XCH_4_ in South Korea was 1917 ppb, while the minimum value was 1704 ppb. The TROPOMI XCH_4_ over South Korea has not yet been validated with the atmospheric measurements. However, there is a possibility of underestimation, as studied at a Japanese site (Lorente et al., 2021), and therefore we try to understand the characteristics of methane distributions and the related emissions associated with higher methane levels.

**Fig. 4:**
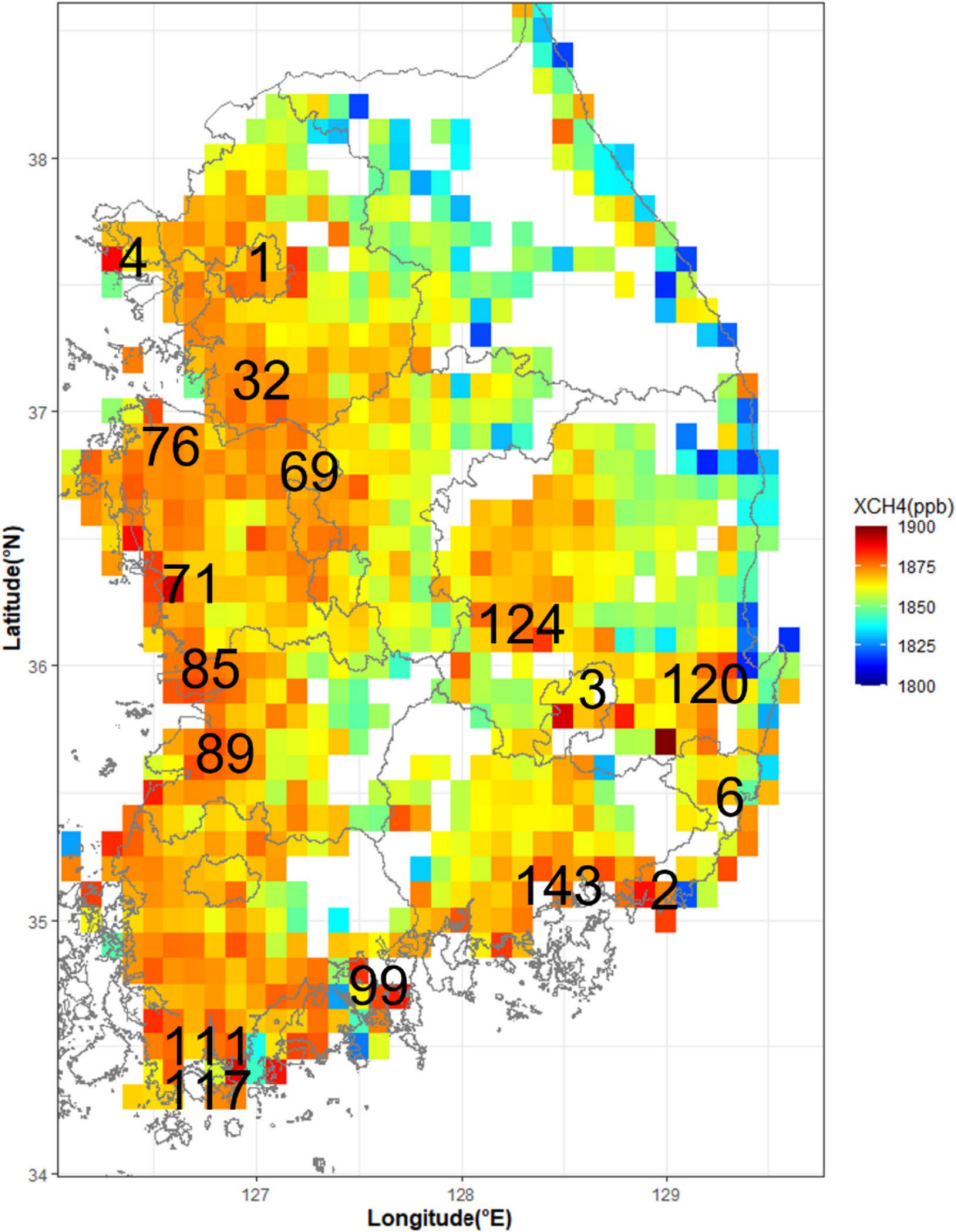
The distribution of average XCH_4_ concentrations in South Korea (from August 2019 to July 2020). The numbers denote the administrative districts with high methane concentrations described in Figure S-1 and Table S-2

When it comes to the spatial distribution of XCH_4_, higher concentrations (> 1870 ppb) were observed in the western coastal region such as Incheon (4), Hwaseong-si (32), Haenam-gun (111), and Wando-gun (117) (Fig. 4). 1870 ppb is equivalent to the 70th percentile of total XCH_4_ samples (1105), and the area with those values was set as high concentration I. Additionally, a value of > 1880 ppb, which corresponds to about 95th percentile of all grids, was selected as high concentration II, and those high XCH_4_ were observed in sporadic areas such as Yeosu-si (99), Gunsan-si (85), Daegu (3), and Busan (2) (Fig. 4).

**Fig. 5:**
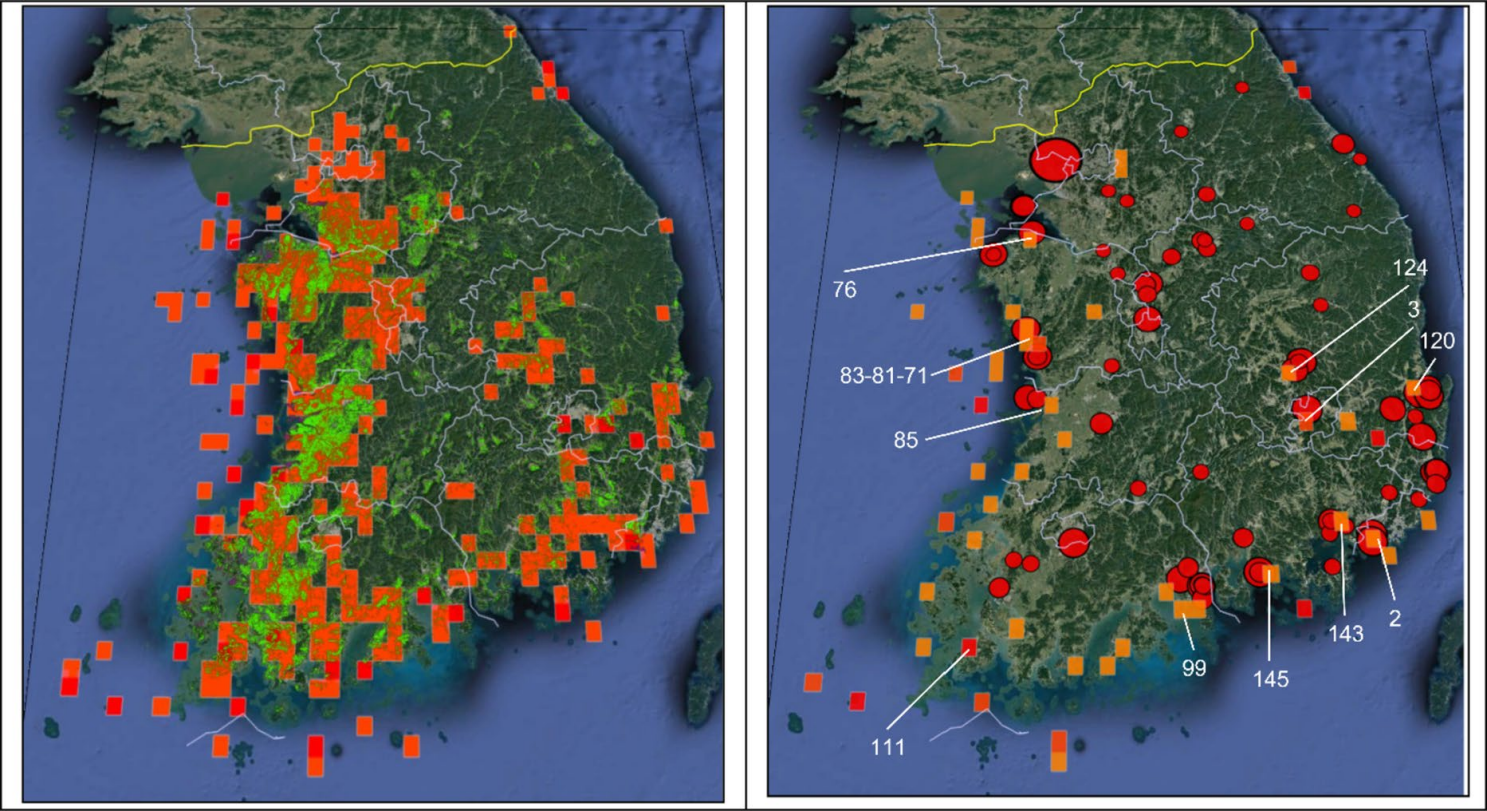
The average concentration of atmospheric methane higher than 1870 ppb with the distribution of rice paddies (left) and the average concentration of atmospheric methane higher than 1880 ppb with the distribution of landfills (right). (The square grids denote relatively methane concentrations, the green spots show rice paddies (From Sub division Land Cover Map: https://egis.me.go.kr), and the red circle denotes a landfill and its size indicates relative amounts of landfill)

When examining the spatial distribution of South Korea’s major methane emission sources concerning the two high-concentration criteria (I and II), the high-concentration area I showed a spatial distribution similar to that of paddy fields, while the high-concentration area II showed a spatial distribution similar to that of industrial complexes and landfills (Fig. 5).

The left panel of Fig. 5 shows grids with methane concentrations greater than 1870 ppb and most of the areas are classified as rice paddies on a land cover map. Among a total of 327 grids that indicate concentrations higher than 1870 ppb, 172 grids (about 70% excluding about 80 grids corresponding to high uncertainty areas such as coastal areas and oceans) coincide with rice paddy areas. As it is known, about 44% of methane emissions in South Korea were attributed to agriculture in 2018 (GIR, 2020), indicating that a large amount of methane is being emitted from rice paddies and contributes to higher methane concentrations over the western and south coastal area.

The right panel of Fig. 5 shows the distribution of landfills in South Korea based on the amount of waste buried and grids with methane concentrations higher than 1880 ppb. Due to the policy that stipulates the installation of landfill facilities where the amount of waste generated and the area of an industrial complex is larger than a certain size in South Korea, the location of landfills can be used to estimate the location of major industrial complexes (An, 2020).

High methane concentrations higher than 1880 ppb over Yeosu-si (99) and Gumi-si (124) are good examples representing industrial complexes and those with a port (Fig. 4). The high concentrations in coastal areas are often with higher uncertainties, but some of them can be also associated with methane emissions from wetlands such as tidal flats, fugitive emissions from fuel production, transportation, and storage processes in ports, and methane slip from incomplete combustion in ships (Ushakov et al., 2019).

We showed the description of the major methane-emitting industries in each corresponding area with high concentrations (> 1880 ppb) in Supplementary Table 1.

### Spatial correlation between XCH_4_ and national emissions

**Fig. 6:**
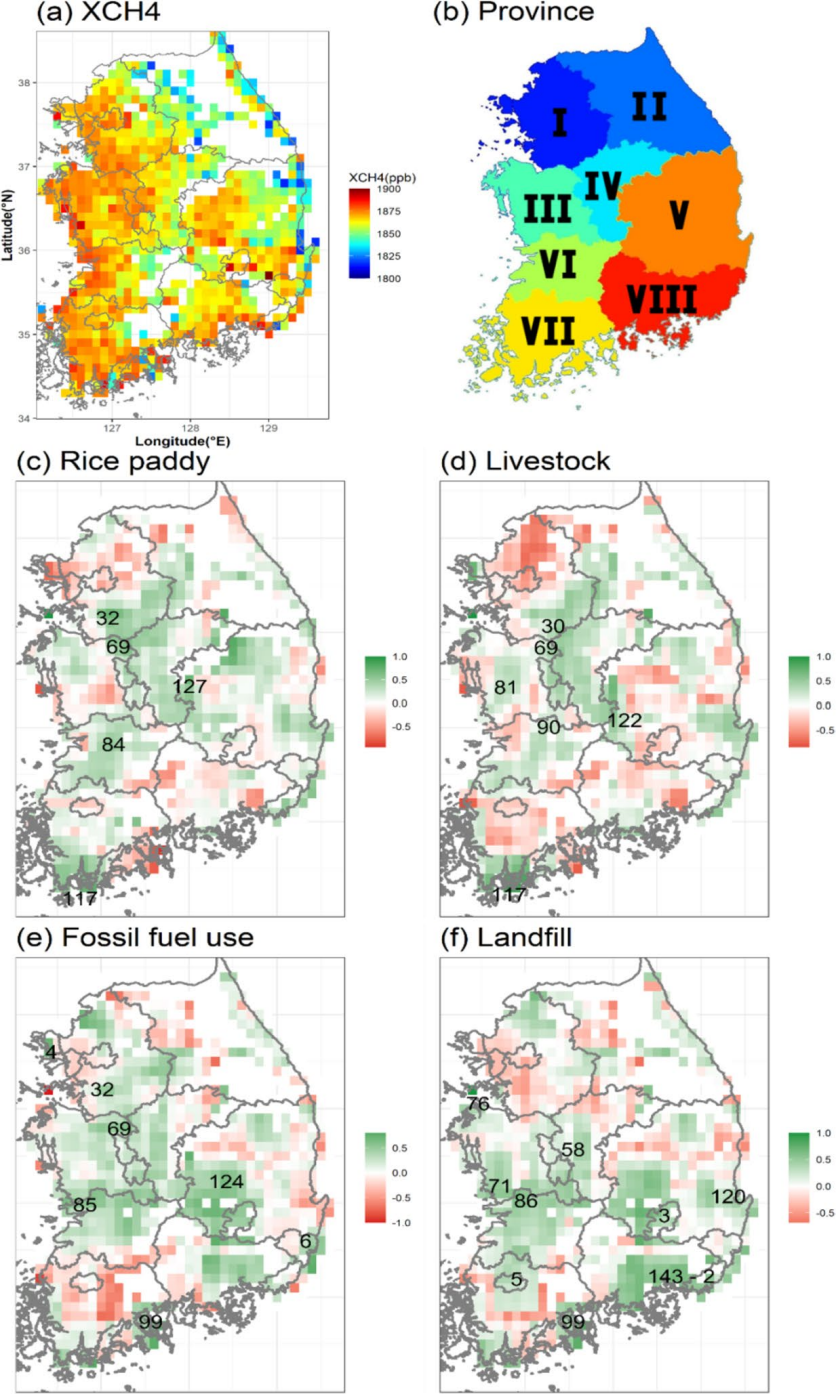
**a** The distributions of annual average concentrations of XCH_4_ (August 2019–July 2020) in South Korea. **b** The locations and numbers of 8 provinces in South Korea. **c** The spatial correlation between XCH_4_ and rice paddy, **d** the same with livestock industries, **e** same with fossil fuel uses, and **f** the same with landfills

To examine the characteristics of high-concentration areas, we classified South Korea into eight regions (Fig. 6b) by local administrative districts. The high concentration in the southern parts of region I showed positive correlations with three emissions: rice paddy, livestock, and fossil fuels, with rice paddy appearing to have higher correlations(*r* = 0.7, Table 1). Region II was excluded from the analysis because sufficient observation data was not available and relatively lower XCH_4_ (mountainous region).

**Table 1:** The high concentration regions of methane by provinces and main sources and correlation coefficients in those regions

Province	Sig_CD	Main source	Correlation coefficient
I	32(Hwaseong-si)	Rice paddy	0.7009
III	71(Boryeong-si)	landfills	0.5045
IV	69(Cheonan-si)	Livestock industry	0.5531
V	3(Daegu)	landfills	0.6958
VI	85(Gunsan-si)	Fossil fuel uses(oil–gas)	0.6317
VII	117(Wando-gun)	Livestock industry	0.8817
VIII	6(Ulsan)	Fossil fuel uses(oil–gas)	0.7690

The high concentrations in regions III, IV, and VI showed weaker positive correlations (*r* < 0.6) with all four emissions, indicating that the complex effects of these four emissions might be at play. Among the four emissions, the high concentration in the northwest region of region III appeared to be influenced greatly by rice paddy (*r*=0.53) and landfill (*r*=0.5), while region IV was mainly affected by livestock (*r*=0.55), and region VI was affected by fossil fuels (*r*=0.63) and landfill (*r*=0.62) (Fig. 6 and Table S-3, S-4, S-5, S-6).

Region VII showed distinct characteristics, with the southwest region indicating positive correlations with rice paddy (*r* = 0.75) and livestock (*r* = 0.89) and the southeast region showing positive correlations with fossil fuels (*r* = 0.77) and landfills (*r* = 0.78). Finally, regions V and VIII showed positive correlations with fossil fuels (*r* = 0.64, 0.8, respectively) and landfills (*r* = 0.7, 0.66, respectively). The high-concentration areas and major emission sources by region, along with their spatial distribution, were analyzed and presented in Table 1.

#### Spatial correlations with rice paddy

Among the four emissions, the rice paddy showed generally higher spatial correlation with methane concentration, indicating positive correlations in the five high-concentration areas with large paddy fields (Fig. 6c). Table S-3 summarizes the correlation coefficients between the rice paddy fields and methane concentrations.

The southern parts of region I showed a high correlation, as it included many areas with large paddy field areas, including the 11th (Hwaseong-si, 32, *r* = 0.70) and 13th (Pyeongtaek-si, 15) largest rice paddy fields in the country, as well as other areas with large paddy fields (29, 36, etc.).

Northwestern region III also showed positive correlations as it included areas with the largest and third-largest paddy fields in the country. The area with the highest correlation (*r* = 0.54) was located in the northeast region III (Cheonan-si, 69), which also showed correlations with other sectors such as livestock and fossil fuels.

In the northwest region V, high correlations were observed in areas with active rice farming (Sangju-si, 127). In the western region (VI), large agricultural land areas also showed high positive correlations, with the highest correlation (*r* = 0.51) observed in the Jeonju-si (84). In Region VII, high correlations were observed due to the influence of large rice paddy fields in the south, with the highest correlation (*r* = 0.75) observed in the southernmost region Wando-gun (117).

#### Spatial correlations with livestock

The correlation analysis between XCH_4_ and the livestock sector showed a similar spatial distribution to those of the rice paddy sector, and positive correlations were observed with some high-concentration areas (Fig. 6d). Table S-4 summarizes the major livestock areas and spatial correlations by region. In the southern parts of Region I, a high correlation was observed in the Anseong-si (30) (*r* = 0.55), which has the second highest number of livestock households and ammonia emissions from manures in the country, and adjacent area Icheon-si (29) has the 4th highest ammonia emissions.

In central Region III, a positive correlation was observed near Yesan-gun (82) (*r* = 0.36) and the adjacent region Hongseong-gun (81), where has the highest ammonia emissions and the fifth highest number of livestock farms in the country.

In Region IV, a positive correlation was observed due to the influence of methane emissions from Cheonan-si (69) (*r* = 0.55), which has many livestock farms. The higher correlations were observed in Gimcheon-si (122) in region V, Wanju-gun (90) in region VI, and Wando-gun (117) (*r* = 0.89) in region VII, where the region’s main industry includes livestock and rice farming. Table S-4 summarizes the correlation coefficients between the livestock fields and methane concentrations.

#### Spatial correlations with fossil fuel use (oil and gas)

The spatial correlation analysis between fossil fuel consumption and XCH_4_ showed different spatial characteristics from rice paddies and livestock fields, and positive correlations were observed in some parts of high-concentration areas (Fig. 6e).

In Region I, higher correlations were found in the northern region with high energy consumption in Paju-si (28) and in the central-western region with high energy consumption in the transportation sector in Namyangju-si (21) (*r* = 0.71). In the southern part of Region I, positive correlations were observed in the vicinity of Hwaseong-si (32) and adjacent Pyeongtaek-si (15) (*r* = 0.50), where there is a high energy use in transportation.

High correlations were also observed in the northern part of Region III, including Cheonan-si (69) with higher vehicular traffic volume, and Cheongju-si (98) nearby (*r* = 0.57). In the southwest part of region V, positive correlations seem to be influenced by the large city (Daegu, 3) (*r* = 0.64) and adjacent Gumi-si (124), where industrial complexes are located.

In the northwest part of region VI, Gunsan-si (85) showed a higher positive correlation due to the higher energy consumption of shipping operations in Gunsan harbor (*r* = 0.63). A high correlation was also observed in the Yeosu-si (99) (*r* = 0.77), where the largest petrochemical complex in Korea is located, and in the eastern part of region VIII, a clear positive correlation was observed in the Ulsan-si (6) (*r* = 0.77) where industrial complexes including steelmaking and chemical plants are located. Table S-5 summarizes the correlation coefficients between fossil fuel use and methane concentrations.

#### Spatial correlations with landfills (wastes)

The spatial correlations of landfills with XCH_4_ showed a distribution similar to that of fossil fuel use, and higher positive correlations were observed in some of the hot spots (Fig. 6f). In the northwestern part of region III, the higher correlation was observed near the area Dangjin-si (76) (*r* = 0.50) where self-landfill sites for large power generation facilities are located, and in Cheongju-si (58) in region IV, where an industrial complex is located with a large amount of landfill, showing positive correlations (*r* = 0.48).

In the southwestern part of region III and northwestern part of region VI, high correlations were observed near areas Boryeong-si (71) and Gunsan-si (85), where large-scale national coal-fired power plants are located (*r* = 0.50, 0.62, respectively).

In region V, the spatial correlation was highest in Daegu-si (3) (*r* = 0.70) where a landfill facility with a large population is located, and higher correlations were also observed near Gumi-si (124) where final waste treatment facilities are located (Fig. 4e).

In Yeosu-si (99), particularly higher correlations (*r* = 0.77) were shown due to the influence of 12 landfill sites located near industrial complexes. In the southern part of region VIII, higher positive correlations were observed in Changwon-si (143) and Pohang-si (120) in the eastern region (*r* = 0.53, 0.66, respectively), where local government landfill facilities, industrial complexes’ landfill sites, and final waste treatment facilities all exist, and also in Busan (2), the largest city in the southeastern Korea where local government landfill facilities and final waste treatment facilities are located. Table S-6 summarizes the correlation coefficients between the landfills and methane concentrations.

### Overall summary of the spatial correlations over higher XCH_4_: source identification of CH_4_ hot spots in South Korea

In order to examine the main sources of higher methane concentrations in Korea, a spatial correlation analysis was performed using TROPOMI XCH_4_ data and methane emissions data from four sectors (rice paddy, livestock industry, fossil fuel use, and waste landfill). To summarize the results, Fig. 4 shows the higher methane concentrations with the descriptions of their contributing sources below.

In the capital city of Seoul (region I in Fig. 6b), there were no clear correlations between any of the four emissions. In contrast, the southern part of region I (Hwaseong-si, 32) showed higher concentrations of methane due to the complex effect of rice paddy, livestock, and fossil fuel with the strongest correlation (*r* = 0.70) found in rice paddies. Area 32 and its surrounding areas are characterized by high amounts of both rice cultivation (12,156 ha) and fossil fuel use in industrial complexes (14,892 kg/year), indicating that the higher methane concentrations were caused by a combination of effects from the multiple emissions.

Dangjin-si (76) has the largest industrial complex in Korea and also the largest rice cultivation area (19,120 ha), and also includes a self-disposal facility for thermal power plants. Therefore, it is believed that the higher methane concentrations in this area were caused by emissions from rice paddy and landfills. Boryeong-si (71) showed a weak correlation with fossil fuels and the highest correlation with landfills. Higher XCH_4_ in Boryeong-si is explained possibly due to the waste disposal and power generation facility.

The inland area of Cheonan-si (69) showed positive correlations with all four emissions.

Gunsan-si (85), which is a port area, showed positive correlations with two emissions: fossil fuel use and landfills. This area has a large amount of fossil fuel usage (8453 kg/year) related to port facilities and energy transportation, and landfill facilities to handle waste generated in national industrial complexes, which contributes to higher methane concentrations.

Gimje-si (89), which has a large cultivated land area, showed positive correlations with all four emissions, but the large rice cultivation area (15,981 ha) appeared to contribute the most to higher methane concentrations.

Gumi-si (124) in the eastern inland and the Daegu (3) showed higher correlations with two emissions: fossil fuel use and landfills. As many industrial complexes are located, it has a large amount of fossil fuel usage (9242 kg/year, 5041 kg/year, respectively) and a large amount of landfill (387,445 kg/year, 30,056 kg/year, respectively) for waste generated in industrial complexes, which is related to higher methane concentrations.

Pohang-si (120) on the southeast coast showed positive correlations in three emissions: rice paddy, livestock industry, and landfills. The positive correlations between rice paddy and the livestock industry are estimated to be influenced by the neighboring Gyeongju-si (121), while the positive correlation in the landfill area is likely due to a large amount of landfill (421,097 kg/year) from landfill facilities that handle waste from the largest domestic steel industrial complex in the region. Unlike other industrial complexes, the correlation due to fossil fuel use was lower, which is likely due to the characteristics of the steel industry that uses coal as its main energy source, which was not included in this study.

Ulsan (6), an industrial complex near the coast, showed positive correlations with all four emissions, and in particular, the amount of fossil fuel usage (117,570 kg/year) used in the petrochemical complex appeared to contribute the most to high methane concentrations.

Changwon-si (143), including a southern industrial complex, showed positive correlations in fossil fuel use and landfills. It is estimated that a large amount of fossil fuel usage (15,160 kg/year) and the landfill (216,691 kg/year) generated by the large industrial complex contribute to high methane concentrations, and it is also adjacent to the Busan (2), which is home to the largest port in the country and can also contribute the high XCH_4_.

Yeosu-si (99), the largest petrochemical complex in Korea, showed positive correlations with fossil fuel use and landfills. The fossil fuel usage (131,844 kg/year) and landfill amount (569,095 kg/year) generated from the petrochemical complex can contribute to the higher methane. The southwest region (Haenam-gun, 111) showed positive correlations with all four emissions, especially in the rice paddies and livestock industry. The III region is known to have the second largest cultivating area (18,467 ha) in Korea. Additionally, it is explained that the higher XCH4 can be influenced by the nearby areas with extensive tidelands (VII area, where 42.5% of Korea’s tidelands are located (KOSIS, 2018)).

## Conclusions and discussions

In this study, we aimed to understand the spatial distribution of methane concentrations in South Korea, utilizing TROPOMI XCH_4_ data from August 2018 to July 2019. Additionally, we tried to identify the main emissions of higher XCH_4_ concentrations through spatial correlation analysis between satellite data and national statistics. The findings may provide useful insights in implementing effective national greenhouse gas mitigation policies.

By utilizing the methane emission indicators prepared here and analyzing spatial correlations at a high resolution of 10 km, we found distinct differences in the sources of higher methane concentrations in terms of their distributions in South Korea: (1) fossil fuel use and landfill sites and (2) rice farming, and livestock areas with some regions with multiple emissions. Furthermore, the application of refined national statistical data in examining spatial correlations with satellite observations has been instrumental in identifying the causes of elevated methane concentrations in various areas. This approach holds significant potential to contribute to the enhancement of South Korea’s official methane emission inventory, which currently does not have detailed spatial information, also addressing challenges that global methane inventories cannot resolve.

During the study period, the average XCH_4_ concentration in South Korea (1863 ppb) was similar to the global average at 1858.4 ppb, but from June to October, the monthly concentrations were comparatively higher due to the prevalence of rice paddies and wetlands in the region. Notably, regions with methane concentrations exceeding 1870 ppb were predominantly located in the western regions, which are dominated by rice paddies, while areas with concentrations above 1880 ppb showed similar distributions to those of large ports or industrial complexes.

Besides the characteristics of rice paddies and wetlands, this work can also explain higher XCH_4_ with energy consumption (fossil fuel use) and waste disposal (landfills), implying the need for regional methane reduction efforts. In the rice paddy and livestock sectors, it is necessary to continuously promote more sustainable agriculture that can minimize methane emissions. This may require long-term policies, as the Ministry of Agriculture, Food and Rural Affairs has recently proposed a 30% reduction by 2030 in greenhouse gas emissions in the agricultural sector, and its implementation is needed.

For city areas and industrial complexes, methane emissions from fossil fuel use and landfills show similar spatial distributions, so it is necessary for local governments to establish reduction measures for both sectors. In particular, low-carbon policies for inland cities such as Cheonan-si (69), Cheongju-si (58), and Daegu (3), as well as Gunsan-si (85) and Yeosu-si (99), and coastal cities in the southeast region such as Busan (2) and Changwon-si (143), are crucial in mitigating national methane emissions.

The assessment of national methane emissions through spatial correlations does not incorporate other physicochemical factors, such as atmospheric transport, that influence methane concentrations. That is the main caveat of the study.

This study is also partly limited by the availability of national methane emissions data. However, the inclusion of additional data sources, such as national coal usage, fugitive emissions in urban areas, and natural methane emissions, could provide more reliable details on regional contributions and potential mitigation measures. Furthermore, gathering information on finer-scale methane emissions, such as city sewerage emissions, through urban-scale measurement projects would be beneficial. Additionally, developing tools to implement urban-scale policies would be necessary to reduce city-level methane emissions.

Finally, the spatial correlation analysis with satellite data conducted in this study proves highly useful in understanding and validating national methane emission information. This is particularly beneficial in cases like Korea, where spatial information on methane emissions is limited or where there is a high likelihood of unidentified emission sources.

### Supplementary Information

Below is the link to the electronic supplementary material.

Supplementary file1 (DOCX 417 KB)

## Data Availability

The data in this study are available upon reasonable request to the corresponding author.
