# Supplementary material for: Evaluation of Korean methane emission sources with satellite retrievals by spatial correlation analysis
Source: Environ Monit Assess. 2024 Feb 22;196(3):296. doi: 10.1007/s10661-024-12449-w (PMC10884166; doi:10.1007/s10661-024-12449-w)
Supplement: Supplementary file 1 — Supplementary file1 (DOCX 417 KB) [file 10661_2024_12449_MOESM1_ESM.docx]

Figure S-1. The administrative districts of South Korea designated by numbers and the names are in Table S-2.


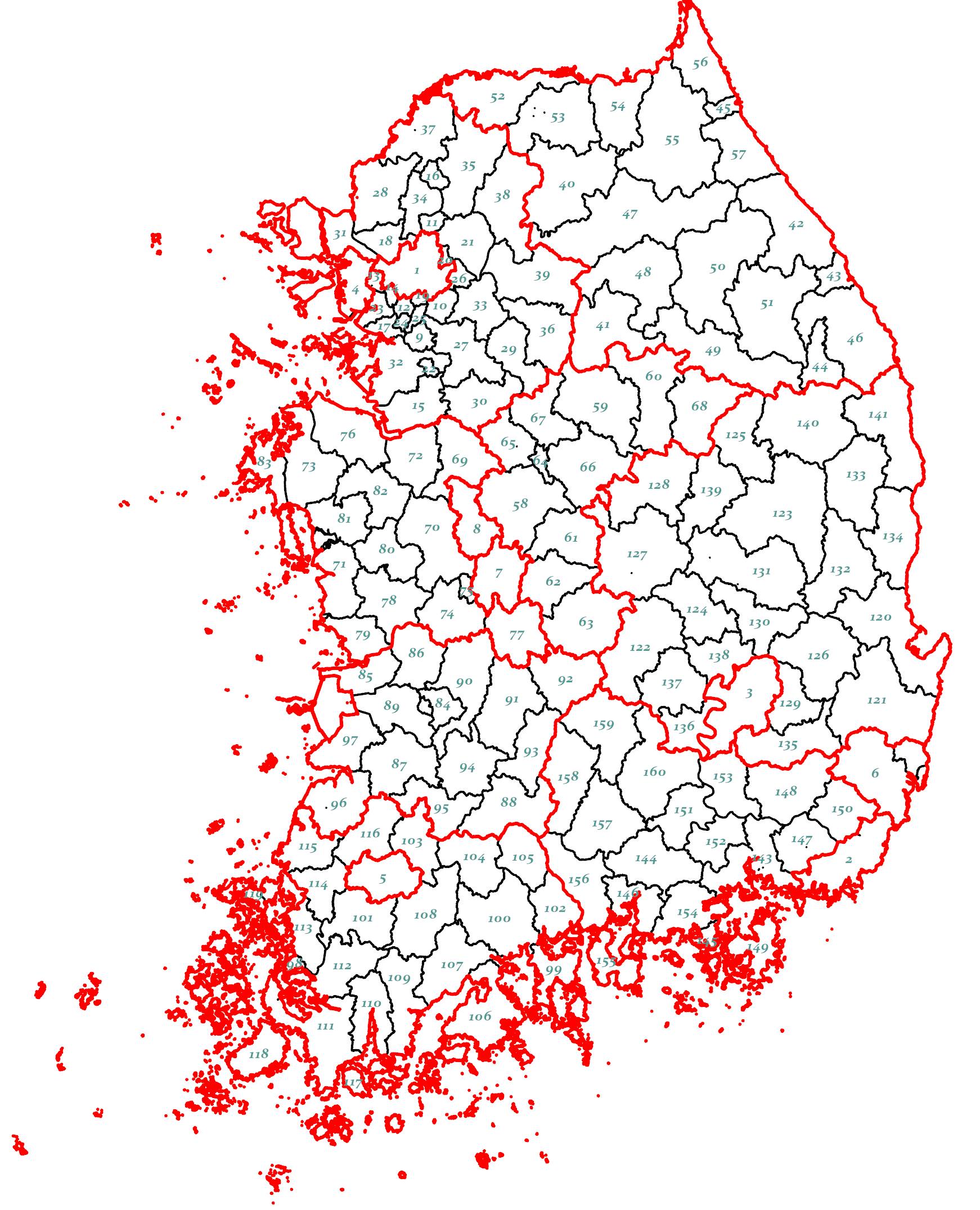


Table S-1. The areas of average concentration of atmospheric methane higher than 1880ppb and the industries which are estimated to be sources of methane emissions in those area.

| Sig_CD | Main industries or characteristics |
| --- | --- |
| 76(Dangjin-si) | Seok-mun national industrial complex (chemical, steel industries, etc.)/ rice paddy area and production volume ranked first in the South Korea |
| 83(Taean-gun)-81(Hongseong-gun)-71(Boryeong-si) | Tideland(coastal wetland) and fishing industry area |
| 124(Gumi-si) | Gumi national industrial complex (electronics, textile industry etc.) |
| 3(Daegu) | Daegu national industrial complex (mechanical metal, automotive industries, etc.) |
| 85(Gunsan-si)-89(Gimje-si) | Gunsan port, industrial complexes (automotive, container industries, etc.)/ rice paddy area and production volume ranked fourth in the South Korea |
| 99(Yeosu-si) | Yeosu, the largest petrochemical industrial complex in South Korea |
| 111(Haenam-gun) | Tideland(coastal wetland) and rice production volume ranked second in the South Korea |
| 120(Pohang-si) | Pohang, The largest steel industrial complex in South Korea |
| 143(Changwon-si) | Changwon national industrial complex (mechanical and electronic industries, etc) |
| 2(Busan) | Busan, The largest trading port in South Korea |
| 145(Tongyeong-si) | Tongyeng, LNG Production Base |

Table S-2. The administrative districts name and code of South Korea

| Sig_CD | Region | Sig_CD | Region | Sig_CD | Region | Sig_CD | Region |
| --- | --- | --- | --- | --- | --- | --- | --- |
| 1 | Seoul | 41 | Wonju-si | 81 | Hongseong-sun | 121 | Gyeongju-si |
| 2 | Busan | 42 | Gangneung-si | 82 | Yesan-sun | 122 | Gimcheon-si |
| 3 | Daegu | 43 | Donghae-si | 83 | Taean-gun | 123 | Andong-si |
| 4 | Incheon | 44 | Taebaek-si | 84 | Jeonju-si | 124 | Gumi-si |
| 5 | Gwangju | 45 | Sokcho-si | 85 | Gunsan-si | 125 | Yeongju-si |
| 6 | Ulsan | 46 | Sancheok-si | 86 | Iksan-si | 126 | Yeongcheon-si |
| 7 | Daejeon | 47 | Hongcheon-gun | 87 | Jeongup-si | 127 | Sangju-si |
| 8 | Sejong | 48 | Hoengseong-gun | 88 | Namwon-si | 128 | Mungyeong-si |
| 9 | Suwon-si | 49 | Yeongwol-gun | 89 | Gimje-si | 129 | Gyeongsan-si |
| 10 | Seongnam-si | 50 | Pyeongchang-gun | 90 | Wanju-gun | 130 | Gunwi-gun |
| 11 | Uijeongbu-si | 51 | Jeongseon-gun | 91 | Jinan-gun | 131 | Uiseong-gun |
| 12 | Anyang-si | 52 | Cheorwon-gun | 92 | Muju-gun | 132 | Cheongsong-gun |
| 13 | Bucheon-si | 53 | Hwacheon-gun | 93 | Jangsu-gun | 133 | Yeongyang-gun |
| 14 | Gwangmyeong-si | 54 | Yanggu-gun | 94 | Imsil-gun | 134 | Yeongdeok-gun |
| 15 | Pyeongtaek-si | 55 | Inje-gun | 95 | Sunchang-gun | 135 | Cheongdo-gun |
| 16 | Dongducheon-si | 56 | Goseong-gun | 96 | Gochang-gun | 136 | Goryeong-gun |
| 17 | Ansan-si | 57 | Yangyang-gun | 97 | Buan-gun | 137 | Seongju-gun |
| 18 | Goyang-si | 58 | Cheongju-si | 98 | Mokpo-gun | 138 | Chilgok-gun |
| 19 | Gwacheon-si | 59 | Chungju-si | 99 | Yeosu-si | 139 | Yecheon-gun |
| 20 | Guri-si | 60 | Jecheon-si | 100 | Suncheon-si | 140 | Bonghwa-gun |
| 21 | Namyangju-si | 61 | Boeun-gun | 101 | Naju-si | 141 | Uljin-gun |
| 22 | Osan-si | 62 | Okcheon-gun | 102 | Gwangyang-si | 142 | Ulleung-gun |
| 23 | Siheung-si | 63 | Yeongdong-gun | 103 | Damyang-gun | 143 | Changwon-si |
| 24 | Gunpo-si | 64 | Jeungpyeong-gun | 104 | Gokseong-gun | 144 | Jinju-si |
| 25 | Uiwang-si | 65 | Jincheon-gun | 105 | Gurye-gun | 145 | Tongyeong-si |
| 26 | Hanam-si | 66 | Goesan-gun | 106 | Goheung-gun | 146 | Sacheon-si |
| 27 | Yongin-si | 67 | Eumseong-gun | 107 | Boseong-gun | 147 | Gimhae-si |
| 28 | Paju-si | 68 | Danyang-gun | 108 | Hwasun-gun | 148 | Miryang-si |
| 29 | Icheon-si | 69 | Cheonan-si | 109 | Jangheung-gun | 149 | Geoje-si |
| 30 | Anseong-si | 70 | Gongju-si | 110 | Gangjin-gun | 150 | Yangsan-si |
| 31 | Gimpo-si | 71 | Boryeong-si | 111 | Haenam-gun | 151 | Uiryeong-gun |
| 32 | Hwaseong-si | 72 | Asan-si | 112 | Yeongam-gun | 152 | Haman-gun |
| 33 | Gwangju-si | 73 | Seosan-si | 113 | Muan-gun | 153 | Changnyeong-gun |
| 34 | Yangju-si | 74 | Nonsan-si | 114 | Hampyeong-gun | 154 | Goseong-sun |
| 35 | Pocheon-si | 75 | Gyeryong-si | 115 | Yeonggwang-gun | 155 | Namhae-gun |
| 36 | Yeoju-si | 76 | Dangjin-si | 116 | Jangseong-gun | 156 | Hadong-gun |
| 37 | Yeoncheon-gun | 77 | Geumsan-si | 117 | Wando-gun | 157 | Sancheong-gun |
| 38 | Gapyeong-gun | 78 | Buyeo-sun | 118 | Jindo-gun | 158 | Hamyang-gun |
| 39 | Yangpyeong-gun | 79 | Seocheon-gun | 119 | Shinan-gun | 159 | Geochang-gun |
| 40 | Chuncheon-si | 80 | Cheongyang-sun | 120 | Pohang-si | 160 | Hapcheon-gun |

Table S-3. The major rice-cultivation areas by province and the highest correlation coefficient in those areas

| Province | Sig_CD(Region) | Rice paddy area(ha) | Correlation coefficient |
| --- | --- | --- | --- |
| Ⅰ | 32(Hwaseong-si) | 12,156 | 0.7009(32, Hwaseong-si) |
|  | 15(Pyeongtaek-si) | 12,088 |  |
|  | 29(Icheon-si) | 8,188 |  |
|  | 36(Yeoju-si) | 6,960 |  |
| Ⅲ | 76(Dangjin-si) | 19,120 | 0.5433(69, Cheonan-si) |
|  | 73(Seosan-si) | 18,352 |  |
| Ⅴ | 127(Sangju-si) | 12,013 | 0.7036(127, Sangju-si) |
|  | 139(Yecheon-gun) | 9,413 |  |
| Ⅵ | 89(Gimje-si) | 16,761 | 0.5076(84, Jeonju-si) |
|  | 86(Iksan-si) | 15,981 |  |
|  | 87(Jeongup-si) | 13,291 |  |
| Ⅶ | 111(Haenam-gun) | 18,467 | 0.7538(117, Wando-gun) |
|  | 112(Yeongam-sun) | 14,821 |  |

Table S-4. The major livestock industry areas by province and the highest correlation coefficient in those areas

| Province | Sig_CD(Region) | NH3 in the livestock sector (T/yr) | Correlation |
| --- | --- | --- | --- |
| Ⅰ | 30(Anseong-si) | 5,348 | 0.5528(30, Anseong-si) |
|  | 29(Icheon-si) | 4,977 |  |
| Ⅲ | 81(Hongseong-gun) | 7,544 | 0.3599(82, Yesan-gun) |
|  | 82(Yesan-gun) | 4,098 |  |
|  | 76(Dangjin-si) | 4,082 |  |
| Ⅳ | 69(Cheonan-si) | 4,630 | 0.5531(69, Cheonan-si) |
|  | 72(Seosan-si) | 2,699 |  |
|  | 58(Cheongju-si) | 2,678 |  |
|  | 8(Sejong) | 2,422 |  |
| Ⅴ | 122(Gimcheon-si) | 2,656 | 0.5487(122, Gimcheon-si) |
|  | 125(Yeongju-si) | 2,660 |  |
|  | 121(Gyeongju-si) | 3,781 |  |
| Ⅵ | 87(Jeongup-si) | 5,186 | 0.3773(90, Wanju-gun) |
|  | 90(Wanju-gun) | 1,853 |  |
| Ⅶ | 101(naju-si) | 3,728 | 0.8871(117, Wando-gun) |
|  | 113(Muan-gun) | 3,052 |  |
|  | 111(Haenam-gun) | 1,269 |  |
|  | 117(Wando-gun) | 1,858 |  |

Table S-5. The major fossil fuel (Gas-Oil) use areas by province and the highest correlation coefficient in those areas

| Province | Sig_CD(Region) | Fossil fuel(Gas-Oil) use (kg/Yr) | correlation |
| --- | --- | --- | --- |
| Ⅰ | 28(Paju-si) | 6,193 | 0.7081(4, Incheon) |
|  | 21(Namyangju-si) | 7,318 |  |
|  | 4(Incheon seo-gu) | 40,016 |  |
|  | 4(Incheon Jung-gu) | 18,405 |  |
|  | 32(Hwaseong-si) | 14,892 | 0.5035(32, Hwaseong-si) |
|  | 15(Pyeongtaek-si) | 13,171 |  |
|  | 27(Yongin-si) | 11,056 |  |
| Ⅲ | 69(Cheonan-si) | 10,636 | 0.5722(69, Cheonan-si) |
| Ⅳ | 58(Cheongju-si) | 13,190 |  |
| Ⅵ | 85(Gunsan-si) | 8,453 | 0.6317(85, Gunsan-si) |
| Ⅶ | 99(Yeosu-si) | 131,844 | 0.7717(99, Yeosu-si) |
|  | 102(Gwangyang-si) | 25,226 |  |
| Ⅴ | 3(Daegu) | 5,041 | 0.6396(124, Gumi-si) |
|  | 124(Gumi-si) | 9,242 |  |
| Ⅷ | 6(Ulsan Ulju-gun) | 62,375 | 0.7690(6, Ulsan) |
|  | 6(Ulsan nam-gu) | 117,570 |  |
|  | 143(Changwon-si) | 15,160 |  |

Table S-6. The major landfill areas by province and the highest correlation coefficient in those areas

| Province | sigungu_CD | Landfill weight (kg/Yr) | correlation |
| --- | --- | --- | --- |
| Ⅲ | 76(Dangjin-si) | 482,790 | 0.5045(76, Dangjin-si) |
|  | 71(Boryeong-si) | 936,687 |  |
| Ⅳ | 58(Cheongju-si) | 366,257 | 0.4836(58, Cheongju-si) |
| Ⅴ | 3(Daegu) | 301,056 | 0.6958(3, Daegu) |
|  | 124(Gumi-si) | 387,445 |  |
| Ⅵ | 85(Gunsan-si) | 165,439 | 0.6221(86, Iksan-si) |
| Ⅶ | 5(Gwangju) | 236,653 | 0.7764(99, Yeosu) |
|  | 99(Yeosu-si) | 569,095 |  |
| Ⅷ | 120(Pohang-si) | 421,097 | 0.5297(120, Pohang-si) |
|  | 143(Changwon-si) | 216,691 | 0.6625(143, changwon-si) |
|  | 2(Busan) | 530,560 |  |
